# Time to Think Antifungal Resistance Increased Antifungal Resistance Exacerbates the Burden of Fungal Infections Including Resistant Dermatomycoses

**DOI:** 10.20411/pai.v8i2.656

**Published:** 2024-03-05

**Authors:** Thomas S. McCormick, Mahmoud Ghannoum

**Affiliations:** 1 Department of Dermatology, Center for Medical Mycology, Case Western Reserve University, Cleveland, Ohio; 2 University Hospitals Cleveland Medical Center, Cleveland, Ohio

**Keywords:** Antifungal Resistance, Dermatophytosis, Terbinafine Resistance, *Trichophytan indotineae*, Recommendation

## Abstract

Increased antifungal resistance is exacerbating the burden of invasive fungal infections, as well as potentially contributing to the increase in resistant dermatomycoses. In this commentary, we focus on antifungal drug resistance, in contrast to antibacterial resistance. We provide a brief historical perspective on the emergence of antifungal resistance and propose measures for combating this growing health concern. The increase in the incidence of invasive and cutaneous fungal infections parallels advancements in medical interventions, such as immunosuppressive drugs, to manage cancer and reduce organ rejection following transplant. A disturbing relatively new trend in antifungal resistance is the observation of several fungal species that now exhibit multidrug resistance (eg, *Candida auris, Trichophyton indotineae*). Increasing awareness of these multidrug-resistant species is paramount. Therefore, increased education regarding potential fungus-associated infections is needed to address awareness in the general healthcare setting, which may result in a more realistic picture of the prevalence of antifungal-resistant infections.

In addition to education, increased use of diagnostic tests (eg, micro and macro conventional assays or molecular testing) should be routine for healthcare providers facing an unknown fungal infection. Two critical barriers that affect the low rates for Antifungal Susceptibility Testing (AST) are low (or a lack of) sufficient insurance reimbursement rates and the low number of qualified laboratories with the capacity to perform AST. The ultimate aim is to improve the quality of patient care through fungal identification, diagnosis, and, where appropriate, susceptibility testing. Here we propose an all-encompassing call to action to address this emerging challenge.

## INTRODUCTION

The literature and media are replete with articles describing antimicrobial resistance (AMR) and its impact on the healthcare system as well as the global economy. In fact, as a testament to the impact of AMR, the World Economic Forum (WEF) concluded that “arguably the greatest risk … to human health comes in the form of antibiotic-resistant bacteria” [1]. Focusing on bacterial antimicrobial resistance is of utmost importance as infections caused by bacteria are responsible for the vast majority of community and hospital-acquired infections, and the availability of a large number of antibacterial drug classes presents a diverse range of resistance mechanisms to investigate. However, in recent years fungal biologists have started to encounter emerging resistance to antifungals that is currently raising concern and calls for action [2].

Increased antifungal resistance (AR) is exacerbating the burden of invasive fungal infections, as well as potentially contributing to the increase in resistant dermatomycoses. In this commentary, we would like to bring antifungal drug resistance to the forefront, providing a historical perspective on its emergence and propose a call to action that includes: (1) raising awareness of fungal diseases and AR; (2) increasing efforts to discover and develop new antifungals through support of funding for Research & Development (R&D); (3) enhancing surveillance and diagnosis; and (4) implementing public health intervention and changes in clinical practice designed to limit the spread of AR and, importantly, lead to successful management of these infections.

## HISTORY OF ANTIFUNGAL INFECTIONS AND RESISTANCE

Prior to significant clinical advancement in interventional medicine, fungal infections, including severe fungal infections, were observed less commonly. The increase in incidence of invasive and cutaneous fungal infections was brought about by advancements in medical intervention, particularly the introduction of anti-inflammatory steroids and immunosuppressive drugs to manage cancer and reduce organ rejection following transplant. Additionally, the overuse of antibiotics to control bacterial infections, particularly broad-spectrum antibiotics, created a permissive environment for fungal overgrowth, especially *Candida*. Table 1 provides risk factors that predisposed patients to invasive candidiasis as previously summarized by Traboulsi and Ghannoum [3].

**Table 1.** Risk Factors Associated with Invasive Candidiasis

Age
Prolonged length of stay in an ICU
High acute physiology and chronic health evaluation score
Central venous catheter
Parenteral nutrition
Broad spectrum of antibiotics
Prolonged antibiotic use
Malignancy
Neutropenia
Bone marrow transplant recipient
Solid organ transplant recipient
HIV/AIDS
Diabetes mellitus
Liver disease
Hemodialysis within 3 months
Renal failure
Autoimmune disease
Immunosuppressive therapy
Surgery within the last 3 months
*Candida* colonization at multiple sites
Very low birth weight (neonate)
Extensive burns
Malnutrition
Severe pancreatitis

In response to the increased prevalence of fungal infections, efforts to develop new antifungals were expedited and led to the development of several new drug classes. In 1950, nystatin (a member of the polyene class) was discovered, followed by amphotericin B (AmB) of the same class. These were the first efficacious drugs approved for the treatment of life-threatening fungal infections [4, 5]. Due to broad-spectrum activity against different genera of fungi that cause invasive fungal infections (IFIs) (eg, *Candida, Aspergillus*, Mucorales) and the ability to successfully treat serious life-threatening fungal infections, AmB rapidly became the “Gold Standard” by which all subsequent antifungals were compared. Another advantage of AmB brought about by its fungicidal activity was that the development of resistance was slow to occur. However, a main disadvantage to this efficacious drug was infusion-related nephrotoxicity, leading some clinicians to refer to it as “ampho-terrible.” This disadvantage provided the impetus to continue the search for new, less toxic antifungal drugs. The developmental timeline of discovery and approval of new antifungal treatments is summarized in Table 2. Supplemental Table 1 shows the chemical structures alongside the presented information summarized in Table 2.

**Table 2.** Antifungal Development and Approvals for Therapeutics Available in the Market by Various Pharmaceutical Industry Members

Decade	Tradename	Generic name	Approval Date	Manufacturer's Name
**1950s**	Nystatin	mycostatin	Discovery (1950) FDA approval date (1971)	Division of Laboratories and Research; New York State Department of Health
	Amphotericin B	Amphotericin B	Discovery Date (1955)	Squibb Institute for Medical Research
	Ancobon	5-fluorocytosine	Discovery (1957) FDA approval (1971)	Roche
**1960s**	Miconazole	imidazole	Discovery (1969) FDA approval (1974)	Insight Pharmaceuticals
**1970s**	Allylamines Naftifine	Allylamines	Discovery (1977)	Sandoz Research Institute
**1980s**	Grifulvin v	Griseofulvin microcrystalline	06/02/1980	OrthoNeutrogena
**1990s**	Diflucan	Fluconazole	01/29/1990	Pfizer
	Lamisil	terbinafine hydrochloride tablets	05/01/1996	Novartis
	Menatax	butenafine hcl cream	01/01/1997	Viatris
	Sporanox	Itraconazole	03/01/1997	Janssen
	Nizoral	ketoconazole	10/10/1997	Johnson & Johnson
	Ambisome	Amphotericin B liposomal Preparation	08/11/1997	Astellas
	Nystatin	nystatin oral suspension usp	06/25/1998	DL laboratories, Inc
**2000s**	Totrisone	clotrimazole/betamethasone dipropionate cream	12/01/2000	Merck
	Cancidas	caspofungin acetate	01/01/2001	Merck
	Vfend	voriconazole	05/01/2002	Pfizer
	Loprox	Ciclopirox Topical Suspension	08/06/2004	Altana
	Mycamine	Micafungin	03/16/2005	Fujisawa healthcare
	Noxafil	posaconazole	09/15/2006	Merck
	Eraxis	anidulafungin	02/01/2006	Pfizer
**2010s**	Jublia	efinaconazole	06/01/2014	Ortho Dermatologist
	Kerydin	tavaborole	07/01/2014	Anacor
	Cresemba	isavuconazonium sulfate	03/01/2015	Astellas
	Luzu	luliconazole cream	02/22/2018	Valeant
**2020s**	Brexafemme	Ibrexafungerp	06/01/2021	Scynexis
	Vivjoa	Oteseconazole	04/01/2022	Mycovia
	Rezzayo	Rezafungin	03/01/2023	Melinta therapeutics

## EPIDEMIOLOGY OF FUNGAL DRUG RESISTANCE

In 1999, Rice and Ghannoum [6] summarized the status of antifungals and antibacterials by focusing on their modes of action and mechanisms of resistance. At that time, most of the attention was devoted to the study of antibiotic resistance in bacteria, whereas the study of antifungal drug resistance lagged. However, in the last two decades, IFIs are now recognized as serious infections with high associated morbidity and mortality (more than 50% mortality, even with therapy), with a significant clinical impact and cost to our healthcare system. Additionally, while early resistance studies were focused on infections caused by *Candida albicans,* largely due to mucosal disease in patients with HIV, an increase in resistant fungal infections caused by non-*albicans Candida* species (eg, *C. glabrata*) [7], molds including *Aspergillus* [8, 9], *Lomentospora* (formerly *Scedosporium prolificans)* and *Fusarium solani* were noted. An increase in these fungal infections in the immunosuppressed population was accompanied by a simultaneous increase in issues around primary AR [10, 11].

Until recently the typical pattern of antifungal drug resistance was limited to resistance to one antifungal class (ie, while a fungal strain may be resistant to an azole it was susceptible to an echinocandin). This changed in 2009 with the emergence of *C. auris* [12]. The specific emergence of the multidrug-resistant *C. auris* represented a paradigm shift in the way we considered antifungal drug resistance. Although multidrug resistance was confined to antibacterials for years, *C. auris* was a fungal species that exhibited both susceptible and multidrug-resistant strains (to quote Dr. Tom Chiller of the CDC, “it became the fungus that behaves like bacteria”). To confront this emerging health threat, the Centers for Disease Control and Prevention (CDC) strongly encouraged all United States-based laboratories that identified *C. auris* to notify their state/local public health authorities as well as the CDC of a reportable fungal infection.

The recent epidemiology clearly demonstrates that resistance to antifungals represents a serious threat to IFI management. In fact, with the rise of echinocandins as first-line therapy for invasive candidiasis, cancer units and ICUs began to see echinocandin-resistant *Candida* species and, at its peak, one medical center reported 14% of bloodstream isolates with the haploid yeast *C. glabrata* were resistant to echinocandins both *in vitro* and *in vivo* [13]. Finally, the breadth of AR, even in superficial fungal infections, continues to evolve as evidenced by recent reports coming out of India and Japan [14] as well as the United States [15] of resistant *Trichophyton* spp. isolated from cutaneous infections. These discoveries emphasize the need for vigilance, close monitoring of AR patterns, and the development of novel antifungal agents.

As summarized in Table 2, the 1990s presented an era of discovery for many new antifungal agents. The triazole compound fluconazole was introduced in the United States in the year 1990. This compound was less toxic than AmB and demonstrated oral bioavailability as well as intravenous applications. It also demonstrated a broad spectrum of activity against yeast. Given the impressive range of activity and low toxicity, the adoption of fluconazole by physicians was widespread despite that supporting data from controlled trials had not been completed [16, 17]. Interestingly, clinical isolates often exhibit resistance to fluconazole, and *C. krusei* is intrinsically resistant to fluconazole. Other species of *Candida* (eg, *C. glabrata* and *C. parapsilosis*) may be initially susceptible but develop resistance following prolonged treatment times. Thus, resistance among *Candida* sp.*,* including *C. auris* has been discussed in the current literature. In the current work, we focus on the newly described emergence of AR among *Trichophyton* spp., including *T. indotineae.*

## ANTIFUNGAL RESISTANCE IN DERMATOPHYTES

Dermatophytosis (Ringworm) occurs worldwide, with an estimated prevalence of 20,000-25,000 per 100,000 persons (~25%), and cutaneous fungal infections represent the highest percentage of fungal infections globally [18]. In humans, dermatophytosis is primarily caused by fungi such as *T. mentagrophytes, T. tonsurans*, and *T. rubrum*, the main causative agents of cutaneous infections of the feet, scalp, body, and nails.

In the early 1990s, the Ghannoum laboratory began monitoring AR to dermatophytes, which coincided with the R&D of terbinafine for the treatment of onychomycosis. Our laboratory, the Center for Medical Mycology at University Hospitals Cleveland Medical Center (CMC), Cleveland, Ohio, was a central lab for phase 3 clinical trials pivotal to the U.S. Food and Drug Administration (FDA) approval of terbinafine. An integral part of terbinafine FDA approval was collecting and testing strains obtained from patients enrolled in these clinical trials. Of 1,432 patients enrolled in the study, 30 patients remained positive following terbinafine treatment, suggesting that they may exhibit terbinafine resistance.

At that time, there was no standardized method for assessing susceptibility of dermatophytes to antifungals, therefore, the CMC optimized such a method (Norris et al, which was published and later adopted by Clinical Laboratory Standard Institutes [CLSI], previously known as NCCLS) and included it in the CLSI reference method M-38 [19]. Applying this antifungal susceptibility method, we showed that failure of patients to clear *T. rubrum* infections did not result in elevated resistance to either terbinafine or to other available antifungals at the time, including itraconazole, fluconazole, or griseofulvin. This indicates that the failure to clear was not related to the development of drug resistance but rather a failure to respond to terbinafine may be related to host factors.

Our team continued to use the dermatophyte Antifungal Susceptibility Testing (AST) method to monitor AR to terbinafine. In 2003, we reported the first case of *Trichophyton* primary resistance to terbinafine, from a patient with onychomycosis [20]. Further antifungal susceptibility testing of this isolate showed normal susceptibility to clinically available antimycotics including itraconazole, fluconazole, and griseofulvin. However, the isolate was fully cross-resistant to several other squalene epoxidase (target of terbinafine and other allylamines) inhibitors: naftifine, butenafine, tolnafiate, and tolciclate, suggesting a target-specific mechanism of resistance.

Continued monitoring of antifungal susceptibility of dermatophytes was performed at our center on samples received from clinical practice as well as isolates from clinical trials. For many intervening years, isolates with elevated resistance to terbinafine were not found; however, this started to change in 2003 when terbinafine and azole-resistant isolates started to appear [20].

Resistance to itraconazole among *Trichophyton* species obtained from patients with superficial dermatophytes or onychomycosis was reported as early as 1994 [21]. Although reports of resistance to antifungals were relatively rare in the early 2000s, publications describing resistant isolates of *Trichophyton* started to appear from various geographic regions in the world, including the United States, between 2017 and 2023 [22–43].

## EMERGENCE OF *T. INDOTINEAE*

Numerous clinical case reports describing terbinafine-resistant dermatophytosis have now been reported worldwide; however, a very strong nidus of resistance was emerging from India by 2018. Currently, there is a sustained epidemic of dermatophytosis in India and neighboring countries. The predominant causative dermatophyte, *T. mentagrophytes* genotype VIII, now designated *T. indotineae,* has been shown to lead to refractory dermatophytosis [24, 40, 44].

Although infections caused by terbinafine-resistant *T. indotineae* have now been reported worldwide, consensus on the nomenclature of *T. indotineae* remains controversial [34]. These resistant variants have spread into Europe, and terbinafine-resistant *T. indotineae* has been identified in Germany [24], France [45][46], Belgium [47], Switzerland [48], Greece [41], Denmark [49], China, Australia, Canada [50] [36], Vietnam [51], and recently Japan [52] and the United States [53]. This worldwide spread may be due in part to travel between India and affected countries; although, endemic resistance has been noted in people who had not traveled recently.

Our group has now monitored AR in the United States for the past 24 years (1999-present). In 1999, we observed no terbinafine resistance in a large cohort of patients (n=1,432) [54]. In 2003, we were the first to report a case of terbinafine-resistant *T. rubrum* in North America [20]. Follow-on studies by Favre et al using the same resistant isolates investigated the biochemical basis for this resistance and showed reduced squalene epoxidase (SQLE, the main target for terbinafine) activity [55].

Therefore, the authors hypothesized that amino acid substitutions were responsible for terbinafine resistance. This observation was later confirmed by Osborne et al, who characterized an amino acid substitution (L393F) in the SQLE gene of *T. rubrum* in 2005 [56]. Further work by Osborne et al [57] characterized an additional amino acid substitution, F397L, caused by a missense mutation in the SQLE gene. Since then, several substitutions, as well as missense and deletions, have been described to inactivate the SQLE gene, resulting in terbinafine resistance. In 2021, we noted an uptick in the number of terbinafine-resistant dermatophytes (*T. rubrum, T. mentagrophytes*) [58].

Few cases of treatment-resistant dermatophytes have been reported in the United States, although individual clinical case reports showed that resistant cases are found in the United States and Canada [15, 36, 58]. However, due to underreporting, given that oftentimes dermatologists/podiatrists request neither cultures nor AST of the infecting strains, it is likely that the number of antifungal-resistant cases is higher. Indeed, most suspected dermatophyte infections are seen in primary care settings and evaluated by physician assistants, nurse practitioners, or general health-care providers.

Therefore, increased education regarding potential fungus-associated infections is needed to address awareness in the general healthcare setting, which may result in a more realistic picture of the prevalence of antifungal-resistant infections. These gaps in understanding the prevalence of dermatophyte resistance in the United States and the concomitant lack of AST, suggest that improved measures for quantifying and reporting resistant cases are urgently needed.

## RECOMMENDATIONS

To address these gaps, we propose a modular approach to assess the burden of antifungal resistant dermatophyte carriage in the United States using molecular epidemiologic techniques. We propose 4 pertinent research fronts: (1) Epidemiologic surveillance to determine the incidence of cutaneous fungal species across the United States, (2) Increased AST, (3) Molecular Identification, and (4) Characterization of the underlying resistance mechanism using sequencing of internal transcribed spacer (ITS) using ITS1 and ITS4 primers, SQLE gene sequence analysis, and phylogenetic analyses (Table 3).

**Table 3.** A Modular Approach to Assess the Burden of Antifungal-Resistant Dermatophyte Carriage in the United States Using Molecular Epidemiologic Techniques

1. Epidemiologic surveillance
2. Increased Antifungal Susceptibility Testing
3. Molecular Identification
4. Characterize underlying resistance mechanism using sequencing approach

We previously conducted several large epidemiological studies within United States and Canadian academic centers, including a local study in Cleveland, Ohio, focused on *Tinea capitis* among 937 children from 8 Cleveland elementary schools. In this study, the background demographic distribution was 87% Black students, and 13% White, Hispanic, or Asian students [59], to determine the incidence of dermatophyte infection in skin, scalp, and nails [60–62]. In other studies, and in collaboration with these sites and the CDC (epidemiology of onychomycosis), we evaluated the incidence of superficial infections in the United States and abroad [58, 59, 61–63]. Thus, we are calling for another large epidemiologic study to be initiated in the United States focused on AR of dermatophytes.

## RECENT *T. INDOTINEAE* CASES IDENTIFIED IN THE UNITED STATES

The previously noted increase in the number of terbinafine-resistant dermatophytes (*T. rubrum, T. mentagrophytes*) [58] as well as individual case reports showing that resistant cases occurred in the United States and Canada indicate that the growing concern regarding antifungal-resistant dermatophytes has reached North America [15, 36]. More alarming is the high likelihood that these cases are undoubtedly a low estimate of the actual incidence since few healthcare providers request cultures or AST.

The realization that infections caused by *T. indotineae* are starting to spread globally prompted the CDC, in 2022, to issue a Broad Agency Announcement focusing on he Topic Area of Interest to reduce Antimicrobial Resistance in Animals and the Environment ([BAA] 75D301-23-R-72545).

## A CALL TO ACTION TO COMBAT ANTIFUNGAL RESISTANCE

It is clear that the global rise and spread of AR is complicating the treatment of superficial and invasive fungal infections. Thus, it is crucial to find alternative comprehensive approaches to combat clinical resistance that go beyond implementing antifungal stewardship programs and the development of novel antifungals that are effective against both susceptible and resistant fungal strains [64]. In this regard, the World Health Organization (WHO) has outlined 4 all-inclusive broad areas of action that build on and reinforce each other:

1. Raise awareness of fungal diseases and AR.
2. Increase efforts to discover and develop new antifungals through support of funding for R&D.
3. Enhance surveillance and diagnosis.
4. Implement public health intervention.

We concur with this prioritization and outline further steps to incorporate these ideas into meaningful actions.

## RAISING AWARENESS OF FUNGAL DISEASES AND ANTIFUNGAL RESISTANCE

Education on mycology diagnostics and AST is paramount. The need to determine whether a patient has a fungal infection is central to treating the patient successfully. As an example, 50% of nail dystrophy cases are caused by fungi, as other disease conditions could mimic nail infections (eg, psoriasis, cancer, bacterial infections etc.). Thus, it is prudent to determine whether a fungus causes the nail dystrophy by performing diagnostic tests (eg, micro and macro conventional assays or molecular testing). Once a strain is isolated, it is critical to perform AST to identify the appropriate antifungal to prescribe. Failure to diagnose and test the susceptibility of the infecting strain will not only lead to treatment failure but also facilitate the development of resistance.

It is important to emphasize that healthcare providers not over-prescribe combination antifungals-corticosteroids for unspecified rashes, which carries the potential for the development of increased levels of resistance. Additionally, patients themselves must be educated to not overuse or misuse over-the-counter antifungal products, as many “fungal” rashes are often self-treated. Two critical barriers that affect the low rates for AST are low (or a lack of) sufficient insurance reimbursement rates for AST testing and the low number of qualified laboratories in the United States that have the capacity to perform AST.

Increasing awareness begins with educating the next generation of practitioners with the tools to recognize and treat fungal infections. Thus, it will be critical to include and enhance medical mycology courses in medical school curricula as well as the curriculum for all generalists, including physician assistants and nurse practitioners. The ultimate aim is to improve the quality of patient care through fungal identification, diagnosis, and, where appropriate, susceptibility testing. Diagnostic tests, although useful, are best integrated into an overall fungal disease management algorithm. Based on our observation over the last 3 decades, medical students have less than 3 hours of cumulative lectures on medical mycology.

Therefore, it is not surprising that diagnosing and treating fungal infections poses a challenge. The educational aspect regarding fungal burden is even lower in other healthcare specialties. Because skin, nail, and hair samples reside superficially, it is also important to educate practitioners and their supporting staff on how best to collect these samples to avoid contamination by non-pathogens that also reside on these areas. Therefore, improving awareness of fungal infections by expanding educational curricula is an important first step. We need to expand healthcare provider education regarding potential fungal infections so that they start “thinking fungus” earlier.

## RESEARCH AND DEVELOPMENT EFFORTS

Research and development to combat IFIs are needed in 3 areas:

1. Development of novel antifungals that are efficacious and safe, and can be delivered orally, topically, and through intravenous routes, as necessary.
2. Development of novel, rapid point-of-care diagnostic tests (eg, PCR-based, lateral flow tests, etc.) to encourage general practitioners to assess potential fungal infections at the source. Along with this initiative, the diagnostic capacity at clinical microbiology laboratories and access to these facilities should also be expanded.
3. Implementation of a robust antimicrobial resistance surveillance system should be a priority.

To expedite the R&D efforts to develop novel antifungals, it is critical to form public-private partnerships to support the development of new therapies (new antifungal classes, new chemical entities, new targets, safety profile and evaluation of cross-resistance) targeting priority antifungal pathogens. In the late 1980s and 1990s, pharmaceutical companies were vested in developing new antifungals to combat the increase in fungal infections affecting HIV-infected patients. The interest in developing new antifungals subsided beginning around 2010, as heralded by the closing of the Pfizer European R&D headquarters in Sandwich, Kent, UK (where the antifungals fluconazole and voriconazole, two life-saving drugs, were studied and manufactured). Other big pharmaceutical manufacturers followed suit. As a result, the development of new antifungals has transitioned to small biotech companies making antifungal drug development challenging given the current economic realities of bringing any drug to market.

## SURVEILLANCE AND DIAGNOSIS

Diagnosing fungal infections is challenging compared to diagnosing bacterial infections due to the biology of fungi, availability of rapid tests, and the limited number of medical mycology trainees. As an example, a fungal blood culture is often negative despite histological analysis that clearly shows fungal elements in bodily organs. This is, in part, due to the increased adhesion of fungal cells (eg, *Candida albicans*) to tissues, whereas bacterial cells are less likely to bind or have a minimal propensity to do so. The number of reliable rapid diagnostic tests currently marketed for fungi is limited. However, this has started to change in recent years with efforts to develop PCR-based assays, as well as lateral flow assays being developed for targeted fungi, as recently noted in the increased number of companies presenting these types of devices at the 11^th^ annual Trends in Medical Mycology Meeting (October 2023). Finally, the number of trainees aiming to gain experience in medical mycology is limited due to the lack of availability of centers of excellence that provide such training and the lack of federal funding to address this need.

Given the rapidly evolving dermatophyte resistance emerging across the globe, the United States must expand surveillance efforts to inform the clinical and research communities of the prevalence of antifungal-resistant strains of dermatophytes. This effort will require a large nationwide epidemiologic study. Supporting this type of (potentially multicenter) study should be a priority of the CDC as well as the National Institutes of Health (NIH).

Active participation in national and international surveillance systems, such as the Global Antimicrobial Resistance and Use Surveillance System (GLASS) and GLASS-FUNGI initiatives, the Latin American Network for Antimicrobial Resistance Surveillance (ReLAVRA), and the European Antimicrobial Resistance Surveillance Network (EARS-Net) should be a targeted, as the CDC has done with their ongoing interactions with both GLASS and GLASS-FUNGI. We can then begin to utilize surveillance data to understand the burden of invasive fungal diseases and drug resistance and inform public health intervention agencies.

## PUBLIC HEALTH INTERVENTION

More specifically addressing the public health burden caused by fungal diseases will lead to the identification of common focal areas that, when incorporated properly, should lead to enhanced prevention and treatment of fungal disease. Topics such as understanding the relationship of fungal infections with disparities, potential social determinants of health, race and/or ethnicity-specific infections, and sex as a biologic variable should be addressed [65]. In addition, many other socio-demographic factors are likely to influence IFIs, and an improved overall understanding of these factors may help identify disparities and lead to the development of effective strategies. Toward this end, broadening the National Notifiable Disease Surveillance System (NNDSS) to increase fungal pathogens will enhance the national surveillance of emerging fungal threats. Globally, establishing an antifungal-resistant priority within the WHO and CDC research agendas for One Health, as part of the antimicrobial resistance program, would help draw attention to emerging AR threats.

## CONCLUSION

Clearly, AR is a problem that is here to stay, and it is likely to increase as detection methods become more sophisticated. Thus, we advocate several steps to enhance our approach to AR:

1. Promote cooperation between pharmaceutical industries, federal agencies, and philanthropic organizations to establish programs with the objective of developing novel antifungals that can prevent and treat emerging resistant fungal infections.
2. Enhance efforts focused on the development of rapid point of care diagnostic tests that could be supported through targeted NIH-supported research funding opportunities and expanded access to advanced mycology clinical laboratories.
3. Establish enhanced educational programs to increase the number of medical mycologists, perhaps through NIH-supported T32 mechanisms, and provide training to practicing health-care providers regarding fungal infections to improve the management of fungal infections and successfully treat afflicted patients.

## SUPPLEMENTARY DATA

Supplementary materials are available at the *Pathogens and Immunity* website. Supplementary data may be provided by the authors to benefit the reader. Supplementary data are not copyedited and are the sole responsibility of the authors. Questions or comments related to supplementary materials should be addressed to the corresponding author.
