# Supplementary material for: Time to Think Antifungal Resistance Increased Antifungal Resistance Exacerbates the Burden of Fungal Infections Including Resistant Dermatomycoses
Source: Pathog Immun. 2024 Mar 5;8(2):158–76. doi: 10.20411/pai.v8i2.656 (PMC10939368; doi:10.20411/pai.v8i2.656)
Supplement: Supplementary Table 1 [file pai-8-158-s001.pdf]

SUPPLEMENTARY TABLE 1

Supplemental Table 1. Antifungal development and approvals for marketed therapeutics available in the market by various pharmaceutical industry members

**Decade**	**Tradename**	**Generic name**	**Chemical Structure (PubChem)**	**Approval Date**	**Manufacturer's Name**
**1950s**	Nystatin	mycostatin	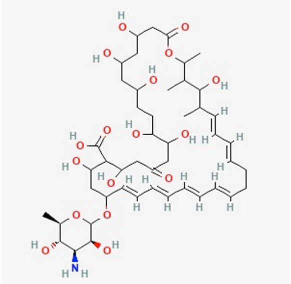	Discovery (1950) FDA approval date (1971)	Division of Laboratories and Research; New York State Department of Health
	Amphotericin B	Amphotericin B	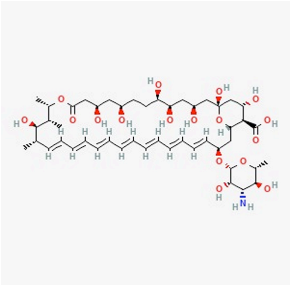	Discovery Date (1955)	Squibb Institute for Medical Research
	Ancobon	5-fluorocytosine	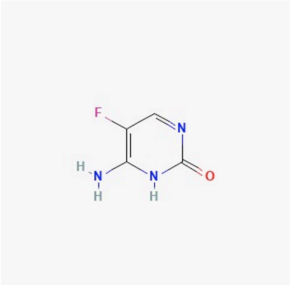	Discovery (1957) FDA approval (1971)	Roche

McCormick T, Ghannoum M. Time to Think Antifungal Resistance : Increased Antifungal Resistance Exacerbates the Burden of Fungal Infections Including Resistant Dermatomycoses. *Pathogens and Immunity*. 2024;8(2):158-176. doi: 10.20411/pai.v8i2.656

**1960s**	Miconazole	imidazole	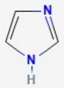	Discovery (1969) FDA approval (1974)	Insight Pharmaceuticals
**1970s**	Naftifine Allylamines	Allylamines	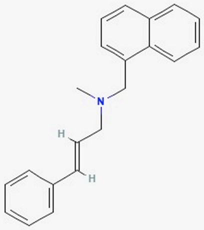	Discovery (1977)	Sandoz Research Institute
**1980s**	Grifulvin v	Griseofulvin microcrystalline	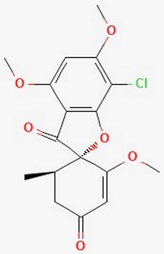	06/02/1980	OrthoNeutrogena
**1990s**	Diflucan	Fluconazole	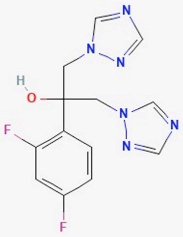	01/29/1990	Pfizer

McCormick T, Ghannoum M. Time to Think Antifungal Resistance : Increased Antifungal Resistance Exacerbates the Burden of Fungal Infections Including Resistant Dermatomycoses. *Pathogens and Immunity*. 2024;8(2):158-176. doi: 10.20411/pai.v8i2.656

PATHOGENS AND IMMUNITY
IN ASSOCIATION WITH CASE WESTERN RESERVE UNIVERSITY AND THE RICHARD J. FASENMYER FOUNDATION

	Lamisil	terbinafine hydrochloride tablets	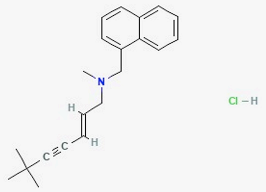	05/01/1996	Novartis
	Menatax	butenafine HCL cream	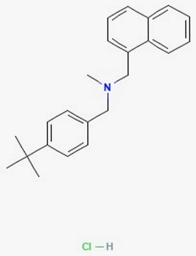	01/01/1997	Viatris
	Sporanox	Itraconazole	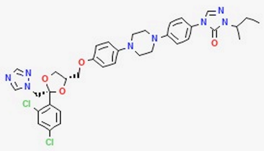	03/01/1997	Janssen
	Nizoral	ketoconazole	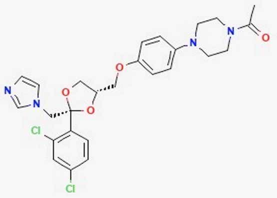	10/10/1997	Johnson & Johnson

McCormick T, Ghannoum M. Time to Think Antifungal Resistance : Increased Antifungal Resistance Exacerbates the Burden of Fungal Infections Including Resistant Dermatomycoses. *Pathogens and Immunity*. 2024;8(2):158-176. doi: 10.20411/pai.v8i2.656

	Ambisome	Amphotericin B liposomal Preparation	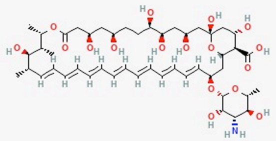	08/11/1997	Astellas
	Nystatin	nystatin oral suspension usp	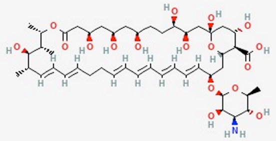	06/25/1998	DL laboratories, Inc.
**2000s**	Lotrisone	clotrimazole/betamethasone dipropionate cream two component cream Component 1 (clotrimazole)	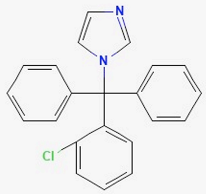	12/01/2000	Merck
		Component 2 (betamethasone dipropionate)	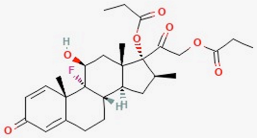		

McCormick T, Ghannoum M. Time to Think Antifungal Resistance : Increased Antifungal Resistance Exacerbates the Burden of Fungal Infections Including Resistant Dermatomycoses. *Pathogens and Immunity*. 2024;8(2):158-176. doi: 10.20411/pai.v8i2.656

	Cancidas	caspofungin acetate	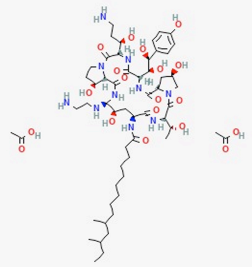	01/01/2001	Merck
	Vfend	voriconazole	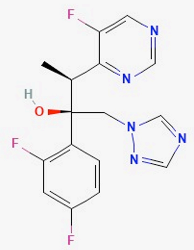	05/01/2002	Pfizer
	Loprox	Ciclopirox Topical Suspension	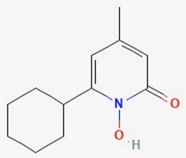	08/06/2004	Altana
	Mycamine	Micafungin	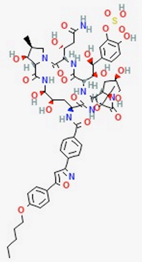	03/16/2005	Fujisawa Healthcare

	Noxafil	posaconazole	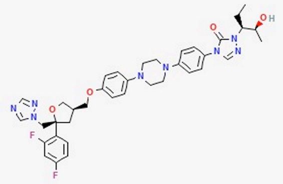	09/15/2006	Merck
	Eraxis	anidulafungin	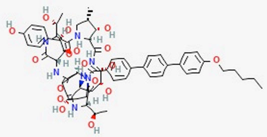	02/01/2006	Pfizer
**2010s**	Jublia	efinaconazole	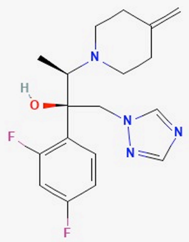	06/01/2014	Ortho Dermatologist
	Kerydin	tavaborole	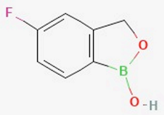	07/01/2014	Anacor

McCormick T, Ghannoum M. Time to Think Antifungal Resistance : Increased Antifungal Resistance Exacerbates the Burden of Fungal Infections Including Resistant Dermatomycoses. *Pathogens and Immunity*. 2024;8(2):158-176. doi: 10.20411/pai.v8i2.656

	Cresemba	isavuconazonium sulfate	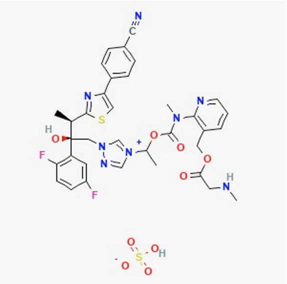	03/01/2015	Astellas
	Luzu	luliconazole cream	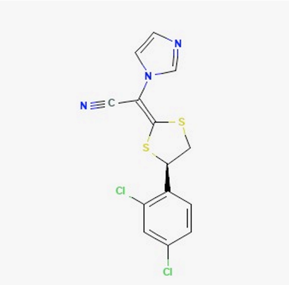	02/22/2018	Valeant
**2020s**	Brexafemme	Ibrexafungerp	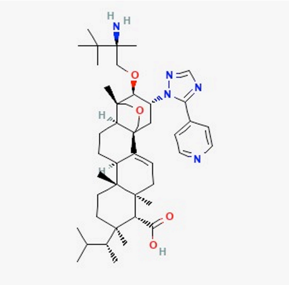	06/01/2021	Scynexis
	Vivjoa	Oteseconazole	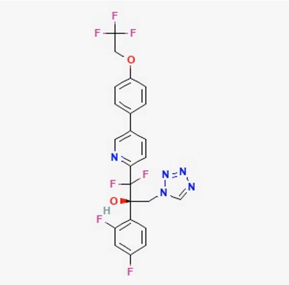	04/01/2022	Mycovia

McCormick T, Ghannoum M. Time to Think Antifungal Resistance : Increased Antifungal Resistance Exacerbates the Burden of Fungal Infections Including Resistant Dermatomycoses. *Pathogens and Immunity*. 2024;8(2):158-176. doi: 10.20411/pai.v8i2.656

	Rezzayo	Rezafungin	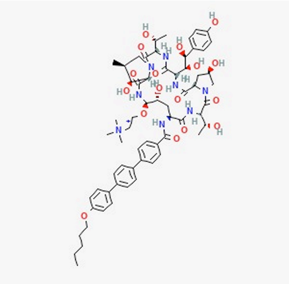	03/01/2023	Melinta therapeutics
